# Effects of video-based natural restorative environments on mental health: a systematic review

**DOI:** 10.1186/s12889-025-21730-7

**Published:** 2025-07-09

**Authors:** Dan Rong Han, Kamal Sabran

**Affiliations:** Department of New Media Design and Technology, Universiti Sains Malaysia, Penang, Malaysia

**Keywords:** Simulated Natural Environment, Restorative Environment, Video-based intervention, Public health, Mental health, New media

## Abstract

**Background:**

Mental health is an essential component of public health. However, many individuals today face serious mental health challenges. Video-based Natural Restorative Environments (VNREs) have shown promising potential in the field of mental health. Despite this, systematic studies specifically addressing VNREs and their effects on mental well-being remain limited. This systematic review aims to 1) assess the current application of VNREs among the general adult population, and 2) examine the specific impact of VNREs on mental health in depth.

**Method:**

This systematic review strictly adhered to PRISMA guidelines and conducted a comprehensive search across six major databases (PsycINFO, PubMed, ProQuest, ScienceDirect, Web of Science, and Taylor & Francis Online) based on the PICOS framework, covering literature published from January 1, 2018, to January 1, 2024. The main inclusion criteria were: 1) studies exploring the relationship between VNREs and mental health; 2) participants being healthy adults; 3) interventions delivered via 2D video. The JBI checklists, along with RoB 2 and ROBINS-I tools, were used to assess study quality and risk of bias. Results were analysed using a narrative synthesis approach.

**Results:**

A total of 26 studies meeting the inclusion criteria were analysed. Current VNREs predominantly depict blue or green spaces, often accompanied by natural soundscapes. Delivered both online and offline across various devices and durations, VNREs demonstrated short-term positive effects on mental health, including improved emotion regulation, stress reduction, and enhanced restorativeness. Additionally, VNREs positively fostered constructive relationships with society and nature, increased energy, improved coping ability, encouraged pro-environmental behaviours, and reduced procrastination. Individual differences and video content are key factors influencing the effectiveness of these interventions.

**Conclusions:**

This review indicates that VNREs offer a flexible and cost-effective approach to mental health intervention. While VNREs cannot fully replicate the experience of real natural environments, they hold significant value in the public health domain. Future research should further examine the effects of intervention design on user experience, with particular attention to how individual differences may moderate intervention outcomes. This review provides a new perspective for developing more effective mental health interventions and offers valuable support for public health practices.

## Introduction

Mental health is an essential component of public health [1]. However, mental health issues have become increasingly prevalent across society, affecting even the general adult population [2]. According to the World Health Organization (WHO), approximately one in eight people worldwide was affected by mental disorders in 2022, with even higher rates in certain regions [3]. These issues primarily manifest as stress [4], depression [5], anxiety [5], and difficulties in emotion regulation [6]. Research indicates that the development of urban green spaces serves as an effective approach to alleviating these mental health issues [7, 8]. However, due to limited economic resources and social factors such as stigmatization and undervaluing of mental health, social inequalities, and gaps in mental health policy and human rights protections, many people's mental health needs remain unmet [3]. There is an urgent need to explore a low-cost, flexible, and spatially accessible approach to mental health intervention to address this growing public health challenge [3].

Restorative environments present one such solution, as they have shown promise in improving mental health [9, 10]. They refer to spaces that facilitate recovery from mental fatigue and stress [11] and are typically rich in natural elements such as forests, water features, and flora [12]. The Attention Restoration Theory suggests that exposure to nature can enhance top-down attentional control [13], while the Stress Reduction Theory suggests that natural environments have powerful stress-relieving effects [14]. The Biophilia Hypothesis further suggests that humans have an innate connection with nature, which fosters profound psychological recovery [15]. Studies have shown that natural restorative environments positively impact emotion regulation [16], stress reduction [14], and cognitive function [17]. Notably, blue spaces (e.g., oceans, lakes, rivers) and green spaces (e.g., parks, forests, gardens) have been widely demonstrated to exert substantial positive effects on mental health [18, 19]. Furthermore, restorative environments are not limited to outdoor settings, indoor spaces like classrooms and healthcare facilities can be designed as restorative environments by incorporating natural elements such as plants [20, 21].

Due to society's growing disconnection from nature, simulated restorative environments are gaining attention for offering psychological benefits comparable to real nature [22–24]. Modern technology has been widely applied to replicate natural scenes, including images [25], two-dimensional (2D) videos [26], and virtual reality (VR) [27]. Two-dimensional (2D) videos, or videos for short, refer to sequences of flat images displayed at a high speed, viewed non-interactively on standard flat screens. Being cost-effective and accessible [28], videos are widely used across various healthcare settings [29]. Research indicates that videos are more effective in reducing stress than static images [30]. While VR technology also holds potential for mental health interventions, its high cost, limited accessibility, potential distractions [31], and health-related concerns [32] restrict its broader application. Moreover, studies have found that 2D videos offer comparable mental health benefits to VR [31, 33, 34], reinforcing the suitability of videos as interventions for enhancing mental health in modern society.

While prior reviews have examined the effects of natural visual stimuli on physiological responses [24] and explored the impacts of simulated nature on human health and cognitive performance [35], there remains a lack of comprehensive analysis specifically focused on the effects of VNREs on mental health. Furthermore, the benefits and applications of VNREs for the general adult population have not yet been thoroughly evaluated. This review aims to address these gaps by 1) investigating the use of VNREs among the general adult population; and 2) analysing their specific impacts and potential benefits on mental health.

## Methods

### Literature sources and search strategy

This systematic review followed the Participants, Interventions, Comparators, Outcomes, and Study Design (PICOS) framework (Table 1) and fulfilled the requirements of the Preferred Reporting Items for Systematic Reviews and Meta-Analysis (PRISMA) guidelines [36]. The literature search covered publications from January 1, 2018, to January 1, 2024. Six databases were systematically searched for relevant titles and abstracts: PsycINFO, PubMed, ProQuest, ScienceDirect, Web of Science, and Taylor & Francis Online. The search strategy is shown in Table 2.

**Table 1:** PICOS

P	The general adult population (excluding studies that focused exclusively on sensitive groups or individuals with specific mental or physical health conditions)
I	2D video of natural restorative environment
C	Different types of natural videos (e.g., different sounds) Other types of videos (e.g., urban videos) Other stimuli (e.g., real nature), Other simulated types (e.g., VR), no intervention (e.g., blank screen)
O	Effect on mental health not measured by biological markers (including aspects related to emotions, behaviour, anxiety, disabling symptoms, constructive relationships, coping ability, stress, and restorativeness, excluding specific mental health disorders such as depression or schizophrenia) and application
S	Randomized controlled trial or quasi-experiments

**Table 2:** Search strategy

Search strategy 1	" video*" OR " video-audio media*" OR " 2D video " OR "videography" OR "film"
Search strategy 2	" restorative environment " OR " natural environment " OR "nature*" OR " therapeutic environment*” OR “therapeutic landscape*” OR “eco-therapy"
Search strategy 3	1 AND 2

Based on the APA Dictionary of Psychology's definition of mental health [37], this review focused on aspects of mental health related to emotions, behaviour, anxiety, disabling symptoms, constructive relationships, coping ability, and stress. Additionally, given the significance of restorativeness in recovering mental health [13], outcomes related to restorativeness were also included in the analysis. Outcomes measured by biological markers were excluded because their complexity could introduce potential confounding variables that were beyond the scope of this review. The final inclusion and exclusion criteria are shown in Table 3. All search results were imported into the EndNote 20 reference management system for easier organisation and subsequent management.

**Table 3:** Inclusion and exclusion criteria

Inclusion criteria	The study focused on video-based natural restorative environments and mental health The participants in the study were sampled from the general adult population The medium of intervention was 2D videos (disseminated through the Internet, mobile devices, or projections, etc.) The study was published in English The study was published in peer-reviewed journals The study was published between January 1, 2018, and January 1, 2024
Exclusion criteria	The study did not include effects on mental health or included only effects on mental health measured by biological markers The medium of intervention includes only forms of VR, CR (Cross-Reality), AR (Augmented Reality), MR (Mixed Reality), 360 videos, video games, and IVE (Immersive virtual environments) The study aimed at a specific group of people that is not representative of the public (e.g. only sensitive groups or individuals with physical or mental conditions were selected, such as the elderly or those with depression) The interventions were not related to nature The study was unpublished or published as a dissertation, a conference article, an abstract, or a review

### Quality assessment checklist

The selected studies primarily consist of randomized controlled trials (RCTs), complemented by quasi-experiments (QEs). Presently, there is no unified checklist to evaluate both research designs simultaneously. To avoid inconsistent results, we adopted a checklist from [38]. This method combines two checklists of The Joanna Briggs Institute's critical appraisal tools for use in JBI Systematic Reviews [39]: one designed for QEs (non-randomized studies) and another for RCTs. The criteria used for quality assessment are shown in Table 4.

**Table 4:** The standard of quality assessment

N	Quality criterion	Explanation
1	What type of research design was applied?	1 = experimental 0.5 = quasi-experimental 0 = not clear (i.e., it is not clearly stated if participants were assigned randomly to the conditions)
2	Was there a control group for the main outcomes?	1 = yes 0 = no
3	Were participants included in any comparisons similar in terms of demographics?	1 = Yes, participants were similar 0.5 = partially, participants differed on some demographics 0 = no or not clear
4	Was the intervention controllable?	1 = yes(unified intervention) 0.5 = (alone or online intervention)
5	Were outcomes measured in a reliable way (validated scales)?	1 = all or just main outcomes were measured using validated scales or the study measured only objective outcomes 0.5 = in part (i.e., one or more scales for measuring main outcomes were created ad hoc) 0 = The study was based on single items as outcomes
6	Was appropriate statistical analysis used?	1 = yes 0.5 = in part 0 = no
7	Were all the mentioned outcomes appropriately reported in the result section?	1 = yes 0.5 = in part
8	Was a power analysis conducted to establish sample size?	1 = Yes, an a priori power analysis was conducted 0.5 = a post hoc power analysis was conducted 0 = no or not clear

A study is classified as “very high quality” if its score is not less than 7, “high quality” if its score is between 5 and 6.5 inclusive, “medium quality” if its score is between 3 and 4.5, and “low quality” if its score is not greater than 2

### Risk of bias

This review adhered to guidelines in the Cochrane Handbook for Systematic Reviews of Interventions [40]. To evaluate the risk of bias, we used the Risk of Bias 2 (RoB 2) tool [41] for RCTs and the Risk of Bias in Non-Randomized Studies of Interventions (ROBINS-I) [42] for QEs in the analysis of included studies. These assessment tools align with checklists used in a similar systematic review [43]. To visually present the bias assessment results, the Robvis tool was used [44].

### Data extraction and data analysis

**Fig. 1:**
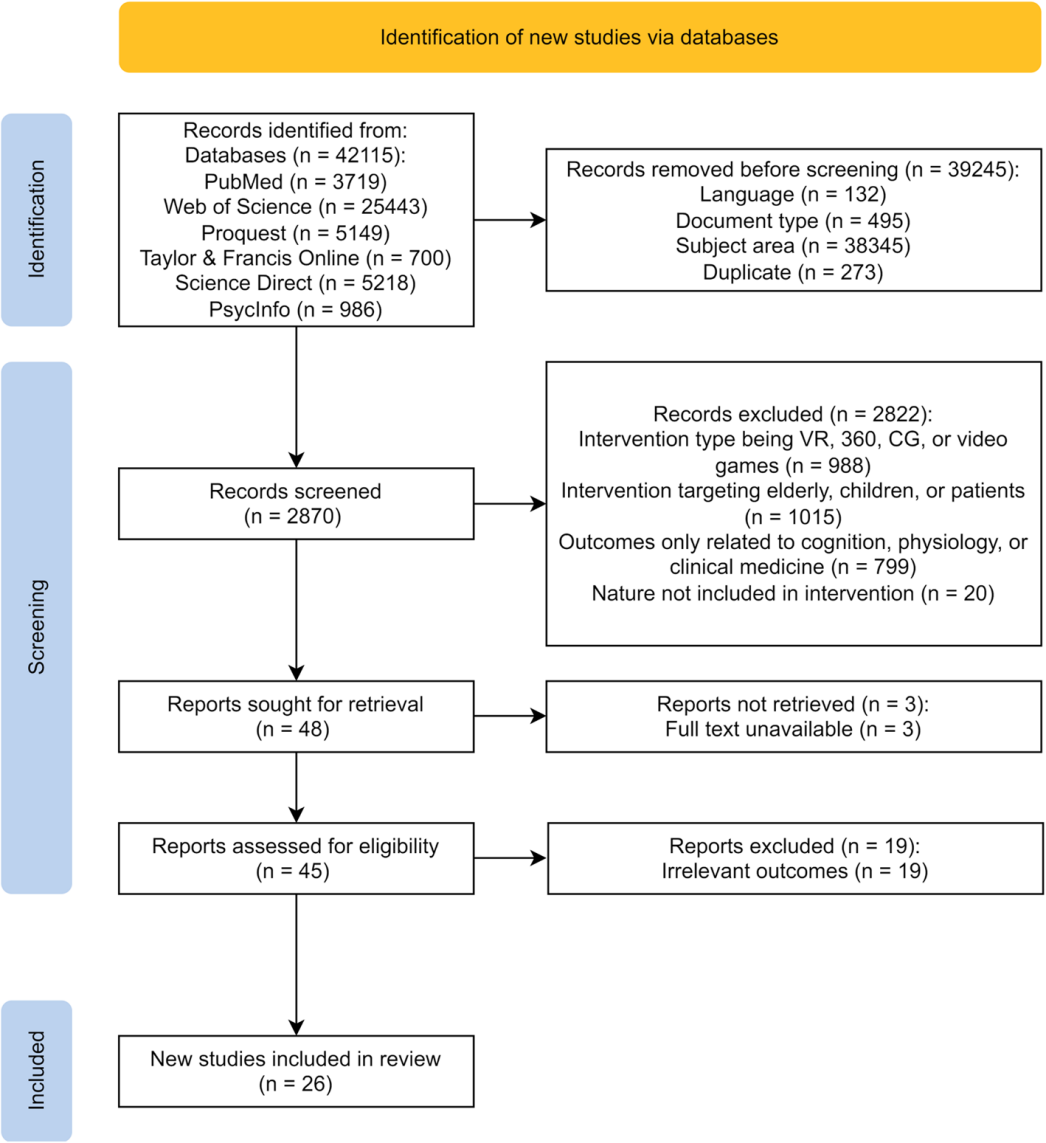
PRISMA flowchart

The initial database search identified 42,115 publications. After filtering by language, document type, research field, and removing duplicates, the number of publications was reduced to 2,870. The authors then screened the titles and abstracts, excluding studies that did not meet the review's criteria for intervention content, type, sample, study outcomes, or research design, as well as publications without accessible full texts, resulting in 45 remaining studies. Based on the inclusion and exclusion criteria, a detailed full-text evaluation was conducted, ultimately including 26 studies. The specific screening process is illustrated in Fig. 1.

All publications were screened independently by DRH. KS was not involved in the initial screening process but double-checked the search strategy and screening procedure, participated in the resolution of any discrepancies, and engaged in discussions regarding study inclusion/exclusion criteria. Disagreements were resolved through consensus. The data extracted by DRH from all studies included author information, publication year, sample details (age, sample size, characteristics, and geographical location of participants), intervention design (duration, devices used and their size, type of intervention, content of videos, and background audio), study design, measurement methods, and key findings. These data were compiled into tables to facilitate systematic comparison and analysis of the research findings (see Table 5, Table 6 and Table 7).

Due to significant heterogeneity in the design of interventions and methods of outcome estimation, a meta-analysis was not feasible. Therefore, this review adopted a narrative synthesis, comparing and analysing the findings of the included studies based on their individual results.

## Results

### Quality

A total of 13 studies were rated as "very high quality," and another 13 were rated as "high quality." No studies were assessed as "moderate quality" or "low quality." All included studies provided clear and comprehensive results. The lowest-scoring item was the power analysis, with an average score of 0.48. Only 11 studies reported a priori power analysis, while three studies reported post hoc power analysis. Additionally, one study was a QE, and the remaining 25 were RCTs. Further details can be found in Table 5.

**Table 5:** The result of quality assessment

Study	Q1	Q2	Q3	Q4	Q5	Q6	Q7	Q8	Total Score	Quality Judgment
Bielinis, E., et al. (2020) [45]	1	1	1	1	1	1	1	1	8	Very high
Brancato, G., et al. (2022) [46]	1	1	0.5	0.5	1	0.5	1	1	6.5	High
Cadogan, E., et. al (2023) [47]	1	1	0.5	0.5	0.5	1	1	1	6.5	High
Diessner, R., et. al (2022) [48]	1	1	0.5	0.5	1	1	1	1	7	Very high
Giachos, D., et al. (2022) [49]	1	1	0.5	0.5	0.5	0.5	1	0.5	5.5	High
Hartanto, A., et al. (2023) [28]	1	1	1	0.5	1	1	1	1	7.5	Very high
Ibanez, L., et al. (2022) [50]	1	1	1	1	1	0.5	1	0	7.5	Very high
Jiang, B., et al. (2021) [51]	1	1	0.5	1	0.5	1	1	0	6	High
Jin, Z, et al. (2022) [52]	1	1	1	1	0.5	1	1	1	7.5	Very high
Kimura, T., et al. (2021) [53]	1	1	1	1	1	1	1	1	8	Very high
Lange, F., et al. (2022) [54]	1	1	0	0.5	1	1	1	1	6.5	High
Lau, S. S. S., et al. (2022) [55]	1	1	1	0.5	0.5	1	1	0	6	High
Li, H., et al. (2021) [56]	1	1	1	1	1	0.5	1	1	7.5	Very high
Li, Z., et al. (2021) [57]	1	1	1	1	1	1	1	0	7	Very high
Meuwese, D., et al. (2021) [58]	1	1	1	1	0.5	1	1	1	7.5	Very high
Meuwese, D., et al. (2021) [59]	1	1	1	1	1	1	1	0	7	Very high
Neale, C., et al. (2021) [60]	1	1	0.5	1	0.5	0.5	1	0	5.5	High
Ojala, A., et al. (2022) [61]	1	1	1	1	1	1	1	0	5.5	High
Reece, R., et al. (2022) [62]	1	1	1	1	1	1	1	0.5	7.5	Very high
Smalley, A. J., et al. (2023) [63]	1	1	0	0.5	1	0.5	1	0	5	High
Sona, B., et al. (2019) [64]	1	1	1	1	1	1	1	0	7	Very high
Suseno, B., et al. (2023) [33]	1	1	1	1	1	1	1	0	7	Very high
van Houwelingen-Snippe, J., et al. (2020) [65]	0.5	1	0.5	0.5	1	0.5	1	0	5	High
Wood, C., et al. (2020) [66]	1	1	1	1	0.5	1	1	0	6.5	High
Yeo, N. L., et al. (2020) [31]	1	1	0.5	1	0.5	0.5	1	1	6.5	High
Zabini, F., et al. (2020) [67]	1	1	0.5	0.5	1	0.5	1	0.5	6	High

### Risk of bias

**Fig. 2:**
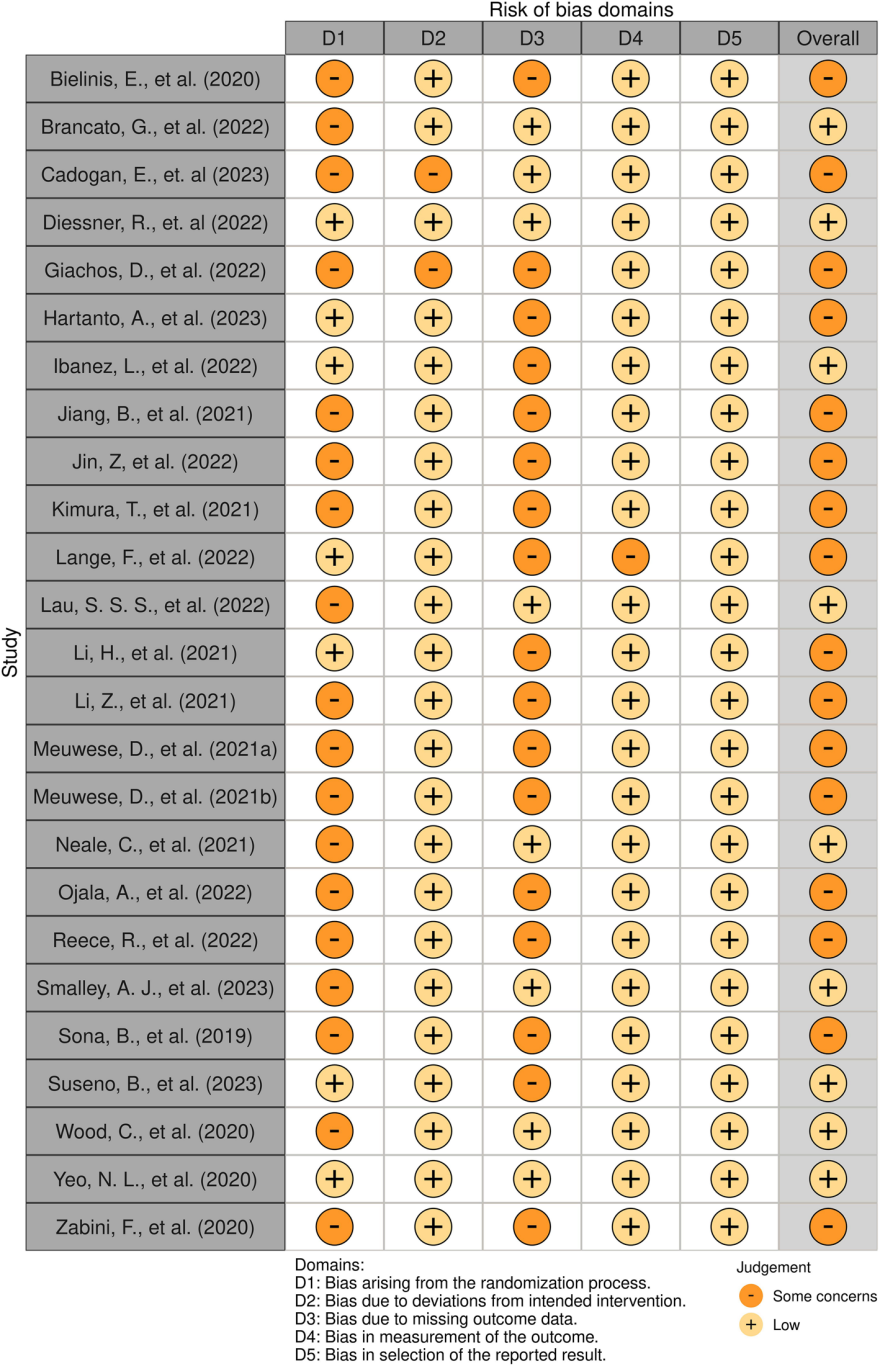
Assessment of risk of bias (RCT)

**Fig. 3:**
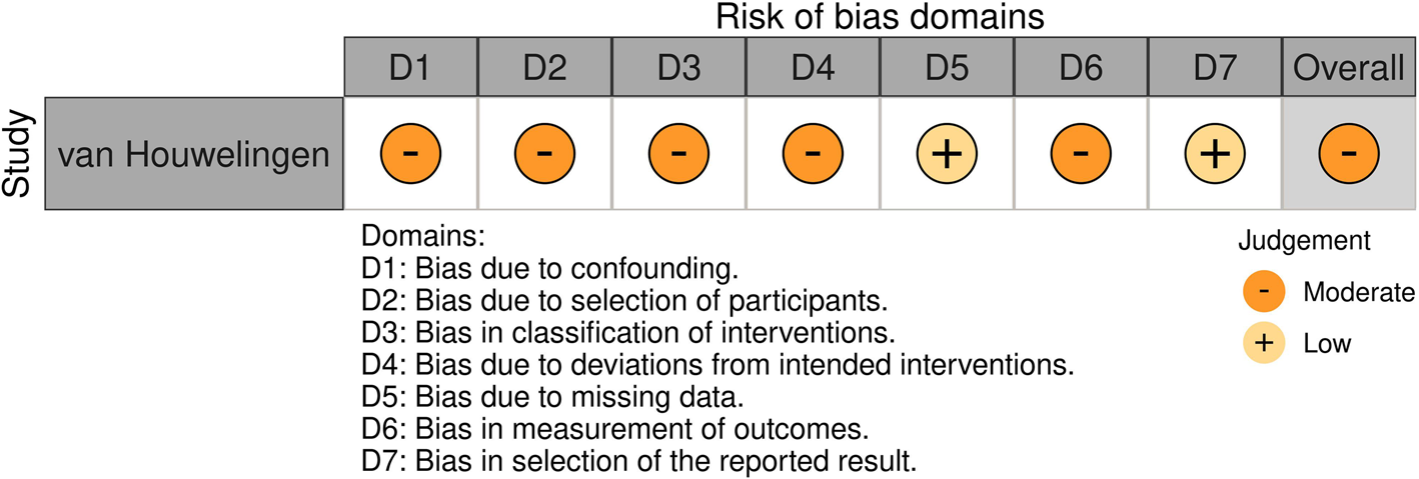
Assessment of risk of bias (QE)

**Fig. 4:**
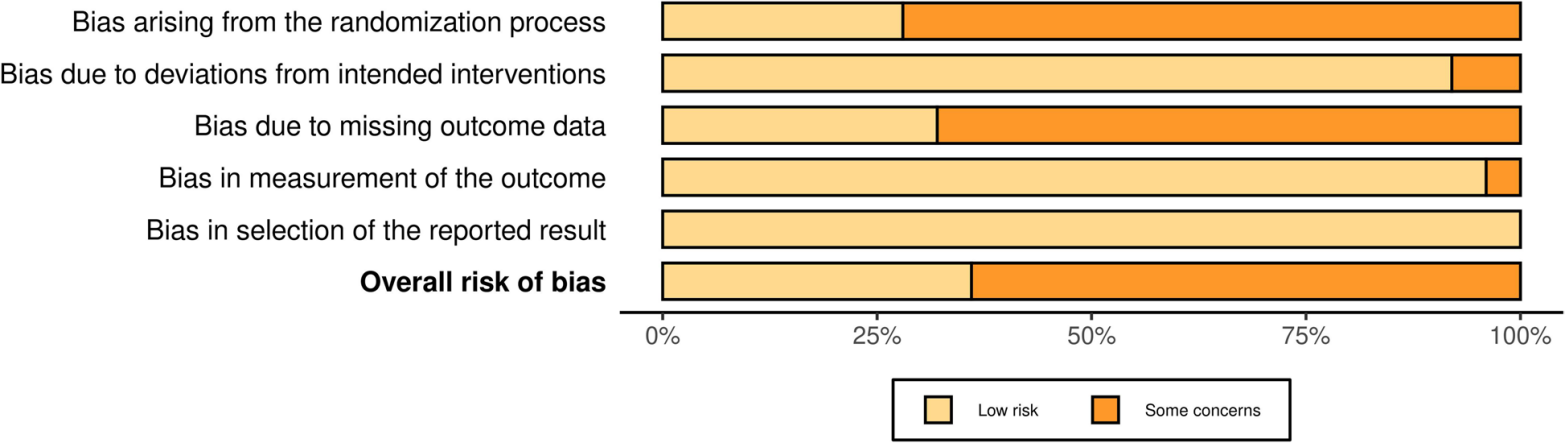
Summary of assessment of risk of bias (RCT)

**Fig. 5:**
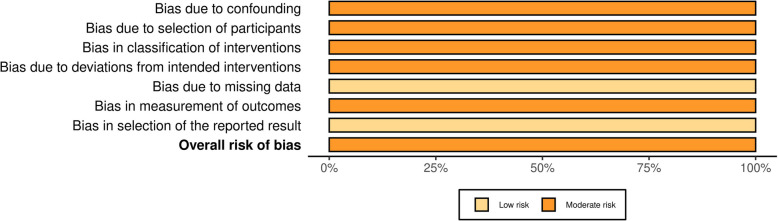
Summary of assessment of risk of bias (QE)

The risk of bias was assessed for 25 studies using the RoB 2 tool. Of these, nine studies were rated as having a "low" risk of bias, and 16 were rated as having a "some concern" risk of bias. None of the studies showed a "high" risk of bias. All studies demonstrated a "low" risk of bias regarding "Selection of Reported Results." However, most studies showed some degree of risk in areas such as "Randomization Process" and "Missing Data." Additionally, one study assessed using the ROBINS-I tool was rated as having a "moderate" risk of bias. Detailed results can be found in Figs. 2 and 3, while summary bar charts are presented in Figs. 4 and 5.

### Sample characteristics

The included studies were conducted across Asia, Europe, and North America. Six studies were conducted in Asia (China: 3, Singapore: 1, Japan: 1, Indonesia: 1), 13 in Europe (UK: 5, Finland: 2, Netherlands: 2, Italy: 1, Germany: 1, France: 1, Belgium: 1), and four in North America (all in the United States). Additionally, three studies used samples from mixed geographic regions: one covering multiple European countries, one covering Europe, Canada, and the United States, and one covering the United States and Hong Kong.

All studies sampled the general adult population using convenience sampling. Eight studies specifically involved university students [28, 45, 53, 55, 58, 59, 62, 64], two studies involved workers [49, 61], and two studies involved individuals during exercise (with one study exclusively involving male participants) [56, 66]. The total sample size was 12,723, with considerable variation in sample sizes across studies, ranging from a minimum of 18 to a maximum of 7,636 participants. The age range was from 18 to 84 years. Additionally, 12 studies did not conduct a power analysis of their sample sizes [33, 50, 51, 55, 57, 59–61, 63–66]. Regarding recruitment methods, 14 studies used offline recruitment [31, 33, 45, 50–53, 56–58, 61, 62, 64, 66], while 11 studies recruited participants via online platforms [28, 46–49, 54, 55, 60, 63, 65, 67]. One study recruited participants both from online and offline [59]. For further details, see Table 6.

### Outcomes

The outcomes were categorized based on the application of VNREs among the general adult population and their effects on mental health. First, outcomes related to the design of VNREs were reported. These included modes of intervention (i.e. online or offline), types of devices used and their sizes, duration of the intervention, video content, and background audio. Next, the outcomes related to mental health effects were reported. These included aspects such as emotions, restorativeness, behaviour, stress and relaxation, constructive relationships, energy, and coping ability. Finally, potential moderating variables that might influence the effectiveness of the interventions were highlighted. A brief overview of the assessment measures used across studies was incorporated throughout the following analysis. For details, refer to Table 6.

**Table 6:** Details of included studies

Authors	Presentation type	Sample specifics	Study design	Measure	Results
Bielinis, E., et. al. (2020) [45]	Offline 55-inch monitor 15 min Green space Natural + Music	42 (mean age 26.24 ± 6.23) College students Finland	QE SEQ BTM	Task: Emotional fatigue induction Emotion: Profile of Mood States (POMS) Restorativeness: Restorative Outcome Scale (ROS) Behaviour(Procrastination): Fluid Procrastination Scale (FPS) Energy: Subjective Vitality Scale (SVS)	1. Reduces short-term procrastination 2. Improves mood, energy and restorativeness
Brancato, G., et. al. (2022) [46]	Online Screen information not provided 15 min Green space Natural sound	202 (age 21–72) USA	RCT SEQ	Emotion: Visual Analog Scale (VAS) State-Trait Anxiety Inventory (STAI-S) Positive Affect Negative Affect Schedule (PANAS) Restorativeness: Perceived Restorativeness Scale (PRS)	1. Pine forest improves mood and happiness 2. Improves restorativeness 3. Farmland has no effects on happiness
Cadogan, E., et. al (2023) [47]	Online Screen information not provided 6min Blue space Green space Natural sound	147 (age 20–80) UK	RCT SEQ	Emotion: Positive Affect Negative Affect Schedule (PANAS)	1. Reduces negative mood 2. The higher the sensory processing sensitivity, the better the effect
Diessner, R., et. al (2022) [48]	Online Screen information not provided 3min Blue space Green space Desert Natural sound	205 (age 22–71) USA	RCT SEQ BTM	Emotion: Elevation Scale four-item scale Awe Scale Behaviour: Intention for Pro-environmental Behaviour (I-PEB)	1. Improves elevation and awe 2. Indirectly promotes intentions for PEB (I-PEB) and donation behaviour
Giachos, D., et. al. (2022) [49]	Online Screen information not provided 3 min each, 30 min in total over the course of 1 week Blue space Green space Desert Natural sound	127 (mean age 36.06 ± 3.13) Workers USA	QE SEQ	Emotion: Hedonic Valence (Pleasantness) Amazon Mechanical Turk (MTurk) Ratings Affective Arousal	1. Increases calmness and valance 2. Increases subjective hedonic experience
Hartanto, A., et. al. (2023) [28]	Online Laptop/desktop 10 min Green space Natural sound	139 College students Singapore	RCT SEQ	Emotion: Positive and Negative Affect Schedule (PANAS)	1. Reduces negative mood 2. Does not increase positive mood
Ibanez, L., et. al. (2022) [50]	Offline Personal computer 12 min Green space Natural sound + Music + Narration	113 (mean age 22.765) France	RCT BTM	Behaviour: Monetary Donation (Eco-Donation) Eco-Action (Recycling Behaviour)	1. Improves pro-environmental behaviour 2. Reinforces pro-environmental behaviour only in individuals with low environmental beliefs
Jiang, B., et. al. (2021) [51]	Offline 22-inch LED 5 min Green space Natural sound Mechanical Sound Traffic Sound Mute	140 USA, Hong Kong	RCT SEQ	Emotion: Multi-Dimensional Mood Questionnaire (MDMQ) Demographic and baseline mood data Stress: Multi-Dimensional Mood Questionnaire (MDMQ)	1. Audiovisual nature videos improve the mood and reduces stress 2. VNREs with mechanical sounds and traffic sounds have negative effects 3. Audiovisual nature videos are generally beneficial to the mood states experienced by native and non-native people
Jin, Z, et. al. (2022) [52]	Offline Screen information not provided 10 min Blue space Green space Natural sound	125 (age 18–32) China	RCT SEQ BTM	Task: Emotional fatigue induction Emotion: Self-Assessment Manikin (SAM) Scale	1. Improves mood and valance (blue space > green space) 2. Depressive symptoms are an influencing factor
Kimura, T., et. al. (2021) [53]	Offline 65-inch LED 5 min Blue space Natural sound	30 (age 20–29) College students Japan	QE SEQ	Emotion: Arousal and Valence Scale	1. The effect of valence was significant 2. No significant effect on arousal
Lange, F., et. al. (2022) [54]	Online Screen information not provided 3 min Green space Music	256 (age 18–77, mean 36.69) Belgian	RCT SEQ BTM	Emotion: Self-report Measures Behaviour: Work for Environmental Protection Task (WEPT)	1. Has positive effect on pro-environmental behaviour 2. Increases happiness and awe
		803 (age 18–84, mean 39.96) UK			1. Has little or no effect on pro-environmental behaviour 2. Increases happiness and awe
Lau, S. S. S., et. al. (2022) [55]	Online Screen information not provided 15 min, 3 times per week, 3 weeks (Total: 135 min) Blue space Green space Natural sound	56 (age above 18) College students Hong Kong	RCT SEQ	Emotion: Profile of Mood State (POMS)-Short Form The Modified Semantic Differential (SD) Well-being: Scale of Psychological wellbeing (SPW) Single item scale Restorativeness: Perceived Restorativeness Scale (PRS) Relationship: Nature Connectedness Scale	1. Increases happiness and comfort but effects disappear two weeks after intervention 2. Increases nature connectedness 3. Has a positive effect on perceived restorativeness two weeks after intervention
Li, H., et. al. (2021) [56]	Offline 88cm x 50cm screen 4 min Green space Natural sound	18 male (mean age 27.94) China	RCT SEQ	Emotion: Feeling Scale (FS)	1. Improves the mood
Li, Z., et. al. (2021) [57]	Offline 75-inch screen 1 min Blue space Green space Natural sound	120 (age 18–35) China	RCT SEQ	Restorativeness: Perceived Restorativeness Soundscape Scale (PRSS)	1. Improves restorativeness (Green space > Blue space)
Meuwese, D., et. al. (2021) [58]	Offline Screen information not provided 4 min Green space Natural sound	198 (age 18–30) College students Netherlands	RCT SEQ BTM	Task: Serious regret (autobiographical recall) Coping ability: State Coping Scale (SCS)	1. Enhances quantity and quality of cognitive coping
		59 (age 18–58) College students Netherlands			1. Enhances quantity of cognitive coping 2. Has no effect on quality of cognitive of coping
		249 (age 18–30) College students Netherlands			1. Enhances quantity and quality of cognitive coping
Meuwese, D., et. al. (2021) [59]	Offline Computer 4 min Green space Natural sound	53 (age 18–37) College student Netherlands	RCT SEQ	Emotion: Zuckerman’s Inventory of Personal Reactions Scale (ZIPERS): Restorativeness: Perceived Restorativeness Scale (PRS) Stress: Profile of Mood States – Short Form (POMS-SF)	1. Reduces stress 2. Has no effect on positive or negative mood 3. Has more benefit when emotionally vulnerable (depressive symptoms) 4. Improves restorativeness
	Online Screen information not provided 4 min Green space Natural sound	196 (age 18–68) general public Netherlands			1. Reduces stress 2. Has no effect on positive mood 3. Improves the negative mood 4. Has more benefit when emotionally vulnerable (depressive symptoms) 5. Improves restorativeness
Neale, C., et. al. (2021) [60]	Online Screen information not provided 3 min—3.5 min Green space Natural sound	200 (age 21–73) USA	RCT SEQ	Emotion: UWIST Mood Adjective Checklist (MACL) Restorativeness: Perceived Restorativeness Scale (PRS) Relationship (community): General Sense of Belonging Scale (GBS) UCLA-3 Loneliness Scale	1. Has positive effect on feelings of belonging and loneliness 2. Improves mood and restorativeness 3. Presence and absence of people in simulation both have no effect on psychological restoration
Ojala, A., et. al. (2022) [61]	Offline 75-inch screen 15 min Green space Blue space Animals Natural sound	39 Workers Finland	QE SEQ	Emotion: Positive and Negative Affect Scale (PANAS) A six-item anxiety scale Restorativeness: Restorative Outcome Scale (ROS) Energy: Subjective Vitality Scale (SVS) Stress: Single-Item Measure of Work Stress	1. Decreases in negative emotion 2. Decreases in positive emotion and energy (Blue < Green) 3. Increases restorativeness and relaxation (sound > silence) 4. Relieves work stress and anxiety
Reece, R., et. al. (2022) [62]	Offline Computer (57 cm × 34 cm) 2 min Blue space Green space Natural sound	28 (age 18–57) College students UK	RCT SEQ BTM	Task: Stressor Videos Emotion: State-Trait Anxiety Inventory (STAI) Stress: University of Wales Institute of Technology Mood Adjective Check List (UWIST MACL)	1. Brings relaxation and calming 2. Reduces anxiety 3. Improves mood (Blue space = Green space)
Smalley, A. J., et. al. (2023) [63]	Online Screen information not provided 3 min Blue space Animal Mute Natural sound Music Music + Natural sound	7636 (age 18–76) UK	RCT SEQ BTM	Task: Stroop Task Attention Network Task (ANT) Emotion: Affective circumplex model Awe and Nostalgia single items scale Restorativeness: Perceived Restorative Potential (PRP)	1. Improves restorativeness and calmness (natural sound > other sound) 2. Increases excitement, awe, and nostalgia (Music + natural sound > other sound)
Sona, B., et. al. (2019) [64]	Offline LED screen (diagonal 165 cm) 15 min Green space Natural sound	122 (mean age 22.69) College students Germany	RCT SEQ BTM	Task: Stroop Task Attention Network Task (ANT) Emotion: Nitsch's Personal State Scale Restorativeness: Perceived Restorativeness Scale (PRS)	1. Promotes recovery of personal resources (mood, fatigue, arousal) 2. Improves restorativeness 3. Combining scents is more conducive to recovery
Suseno, B., et. al. (2023) [33]	Offline 22-inch tab 5 min Blue space Natural sound	28 (mean age 19.8) Indonesia	RCT SEQ BTM	Task: Sing-a-Song Stress Test (SSST) Emotion: State-Trait Anxiety Inventory (STAI)-State subscale Stress: Autonomic Perception Questionnaire (APQ)	1. Reduces anxiety 2. Reduces stress (self-consciousness towards body sensations)
van Houwelingen-Snippe, J., et. al. (2020) [65]	Online Screen information not provided 4 min 6 s Green space Natural sound	1203 Nordic countries, Canada, USA	QE SEQ	Emotion: Awe Experience Scale Relationship: Inclusion of Community in the Self Scale (ICS)(community) Social Aspiration Scale (community) UCLA Loneliness Scale (community) Spatial Presence Experience Scale (nature) Nature Relatedness Scale (nature) Restorativeness: Perceived Restorativeness Scale (PRS)	1. Increases a feeling of connectedness (community) and natural connection 2. Reduces loneliness 2. Relieves feelings of awe and sense of presence 3. Tended nature scenes elicit more social aspirations than wild nature scenes 4. Wild nature scenes elicit more restorativeness than tended nature scenes
Wood, C., et. al. (2020) [66]	Offline Screen information not provided 30 min Green space No record	18 (mean age 32.2 ± 8.5) UK	RCT SEQ BTM	Task: Trier Social Stress Test (TSST) Stress: Stress-Arousal Checklist [68]	1. Reduces stress when doing exercise
Yeo, N. L., et. al. (2020) [31]	Offline 17-inch LED 5 min Blue space Natural sound	96 (age above 18) Europe	RCT SEQ	Emotion: Multidimensional State Boredom Scale (MSBS) Summary of Positive and Negative Experiences (SPANE) Scale Relationship: Inclusion of Nature in Self (INS) Scale(nature)	1. Reduces boredom and negative mood 2. Increases natural connection
Zabini, F., et. al. (2020) [67]	Online Screen information not provided 5 min Green space Natural sound	75 (mean age 47.3) Italy	RCT SEQ	Emotion: State-Trait Anxiety Inventory (STAI) Form Y Sheehan Patient Rated Anxiety Scale (SPRAS) Part II	1. Decreases in anxiety 2. Only short-term effects were observed

#### Design of VNREs

##### Modes of intervention and devices

All interventions included in this review used 2D videos. A total of 14 studies reported the effects of VNREs delivered via various offline devices, including computers, tablets, and public screens [31, 33, 45, 50–53, 56–58, 61, 62, 64, 66]. 10 of these studies explicitly recorded the screen sizes, which ranged from 17 to 75 inches. Most of the offline interventions reported significant positive effects on mental health, including improvements in emotion, stress relief, enhanced restorativeness, and other positive effects. Additionally, 11 studies delivered VNREs through online platforms, leading to uncertainty regarding the types of devices and screen sizes used [28, 46–49, 54, 55, 60, 63, 65, 67]. Most of these studies indicated that online VNRE interventions could effectively improve mental health, such as by promoting PEB, increasing well-being, and enhancing the sense of social connectedness. Notably, one study employed both online and offline interventions [59]. The results showed that offline interventions had no significant effect on participants' positive or negative emotions, while online interventions significantly reduced negative emotions. Overall, no studies explicitly indicated that the mode of intervention (online or offline), device type, or screen size had a substantial impact on the effectiveness of VNRE interventions.

##### Intervention duration

In most studies, the duration of the intervention matched the length of the VNRE videos, ranging from 1 to 30 min. Eight studies used videos that were 10 min or longer [28, 45, 46, 50, 52, 61, 64, 66]. The finding indicated that interventions lasting up to 30 min could significantly reduce stress among participants engaged in physical activities [66]. Meanwhile, 16 studies employed shorter videos, with durations of less than 10 min [31, 33, 47, 48, 51, 53, 54, 56–60, 62, 63, 65, 67]. The finding showed that even a one-minute VNRE could provide relaxing and restorative experiences [57].

Additionally, two studies implemented long-term VNRE interventions [49, 55]. In one study, participants watched one 15-min natural video three times a week over three weeks [55]. Results of this study showed improvements in emotion and a stronger sense of nature connectedness by the end of the intervention, although restorativeness did not significantly change. During a follow-up two weeks later, the improvement in emotion did not persist, but restorativeness significantly increased, and the sense of nature connectedness remained. In another study, participants underwent a one-week intervention where they watched 3-min VNREs multiple times, totalling 30 min [49]. This intervention led to a significant increase in relaxation and positive emotions.

Overall, no studies suggested that the duration of the intervention had a substantial impact on the effectiveness of VNREs.

##### Video content

During the interventions, 14 studies used VNREs that depicted green spaces, such as scenes of forests, meadows, vegetation, lawns, and farmland [28, 45, 46, 50, 51, 54, 56, 58–60, 64–67]. Most of these studies reported positive effects of green-space VNREs on mental health, such as improvements in emotion and behaviour. However, four studies found no significant or even negative effects on mental health, mainly regarding emotion, behaviour, and coping ability [28, 46, 54, 58]. These studies did not explicitly link the negative outcomes or lack of positive effects to the green space itself. One study noted that while forest scenes significantly improved well-being and emotion, farmland scenes had no effect on well-being [46]. Additionally, four studies featured VNREs of blue spaces, which consisted of natural settings such as oceans, rivers, waterfalls, and streams [31, 33, 53, 63]. These studies consistently reported positive effects on mental health, such as improving emotion and reducing stress, from blue-space VNREs.

Eight studies included VNREs featuring both blue and green spaces [47–49, 52, 55, 57, 61, 62]. Most of these studies found that both types of environments promoted mental health by improving emotion and reducing stress. However, Ojala et al. [61] observed a decline in participants' positive emotions in both blue and green spaces, but the decline was less pronounced in blue spaces. Despite this, negative emotions, stress from work, and restorativeness improved across both settings. Besides green and blue spaces, two studies also examined VNREs featuring animal scenes in nature [61, 63]. Both studies reported positive effects on emotion, although they did not specify whether these effects were directly associated with the presence of animal scenes.

##### Background audio

In most studies, VNREs were typically accompanied by natural sounds consistent with the video content, such as birdsong, rustling leaves, and ocean waves. The studies indicated that VNREs with natural sounds effectively improved emotion, enhanced restorativeness, reduced stress, and improved behaviour. Additionally, music was frequently used as background audio in VNREs. Two studies specifically examined the effects of using music alone as the background for VNREs [54, 63]. Smalley et al. [63] reported that VNREs with music alone increased feelings of excitement in participants but did not show positive effects on a sense of awe or nostalgia. In contrast, Lange et al. [54] found that the intervention enhanced well-being and a sense of awe; however, it did not significantly promote PEB.

In addition to music alone, the combination of music with other sounds was also common in VNRE designs. Two studies found that VNREs featuring a mix of music and natural sounds improved behaviour, enhanced restorativeness, increased energy, and improved emotion [45, 63]. Smalley et al. [63] specifically noted that the combination of music and natural sounds increased feelings of excitement. One study also discovered that combining music, natural sounds, and narration about nature helped promote PEB after the intervention [50].

One study used mechanical sounds and traffic noise as background audio and found that these noises led to negative emotions and increased stress [51]. Additionally, three studies tested silent VNREs, showing that, compared to those with natural sounds, silent VNREs were less effective in improving emotion, reducing stress, and enhancing restorativeness [51, 61, 63]. One study reported that silent VNREs had no effect on emotion or restorativeness [63].

#### Effects of VNREs on mental health

Table 7 shows the included studies categorized by outcome and the main results.

**Table 7:** The included studies' outcomes and main results

Outcomes		Studies	Measure	Main Result
Emotion	Positive Emotion	[28, 45, 46, 48, 49, 51–56, 59–65]	Emotion: The Positive Affect Negative Affect Schedule (PANAS)[28, 46, 47, 61]; The Profile of Mood States (POMS)[45, 55, 59] The Amazon Mechanical Turk (MTurk) Ratings [49]; The Self-Assessment Manikin (SAM) Scale [52]; The Modified Semantic Differential (SD) [55]; Anxiety: The State-Trait Anxiety Inventory (STAI) [33, 46, 62, 67]; Awe: The Awe Experience Scale [65]; The Awe Scale [48]; The Awe and Nostalgia Single Items Scale [63]	Most studies showed positive effects, and some studies showed no effects, even negative effects. Results are inconsistent
	Negative Emotion	[28, 31, 33, 47, 59, 61, 62, 67]		Most studies showed positive effects
Restorativeness		[45, 46, 55, 57, 59–61, 63–65]	The Perceived Restorativeness Scale (PRS) [46, 55, 59, 60, 64, 65] The Restorative Outcome Scale (ROS) [45, 61]; The Perceived Restorativeness Soundscape Scale (PRSS)[57]; The Perceived Restorative Potential (PRP) [63]	Most studies showed positive effects
Stress and Relaxation		[33, 51, 59, 61, 62, 66]	Mood: The Multi-Dimensional Mood Questionnaire (MDMQ) [51]; The Profile of Mood States – Short Form (POMS-SF) [59]; The University of Wales Institute of Technology Mood Adjective Checklist (UWIST MACL) [62] Exercise-related stress: The Stress-Arousal Checklist [66, 68] Workplace stress: The Single-Item Measure of Work Stress [61]; Stress-related self-consciousness towards body sensations: The Autonomic Perception Questionnaire (APQ) [33]	All studies showed the positive effect on stress
Behaviour	Procrastination	[45]	The Fluid Procrastination Scale (FPS) [45]	Further studies are needed
	Pro-Environment Behaviour	[48, 50, 54]	The Intention for Pro-environmental Behaviour (I-PEB)[48]; Monetary Donation (Eco-Donation) and Eco-Action (Recycling Behaviour) [50]; The Work for Environmental Protection Task (WEPT)[54]	
Construction Relationship	The Sense of Natural Connectedness	[31, 55, 65]	The Nature Connectedness Scale [55]; The Inclusion of Nature in Self (INS) Scale [31]; The Nature Relatedness Scale [65]	Further studies are needed
	The Sense of Social Connectedness	[60, 65]	The sense of social connectedness: The UCLA Loneliness Scale [60, 65]; Belonging: The General Sense of Belonging Scale (GBS) [60]; Social Aspirations: The Inclusion of Community in the Self Scale (ICS); The Social Aspiration Scale [65]	
Coping Ability		[58]	The State Coping Scale (SCS)[58]	Further studies are needed
Energy		[45, 61]	The Subjective Vitality Scale (SVS)[45, 61]	Results are inconsistent. Further studies are needed

##### Emotion

A total of 20 studies reported changes in emotions after viewing VNREs. These changes included both positive and negative emotions, with some studies focusing on specific feelings such as boredom, anxiety, awe, nostalgia, excitement, elevation, well-being, valence, calmness, comfort, arousal, emotional personal resources, and subjective hedonic experiences. All studies used self-reported questionnaires to measure emotional (Table 7). Notably, two studies conducted mood induction tasks before delivering the VNRE [45, 52].

##### Negative emotions

Eight studies examined the effects of VNREs on negative emotions. Most findings indicated that VNREs offered effective visual stimulation that captured attention, reduced fatigue, and provided calming experiences, significantly improving negative emotions [28, 31, 33, 47, 59, 61, 62, 67]. However, one study that repeated the intervention twice found no effect on negative emotions in the first intervention but observed significant improvement in the second [59].

Some studies addressed specific negative emotions. For example, Yeo et al. [31] discovered that VNREs, even when compared to more immersive virtual natural environments, effectively reduced boredom through visual engagement. Additionally, four studies reported the positive effects of VNREs in reducing anxiety[33, 61, 62, 67]. Among them, Zabini et al. [67] found that even a brief five-minute exposure to green space had a significant short-term effect on reducing anxiety, although no long-term benefits of VNREs in anxiety reduction were observed.

##### Positive emotions

The results on the effects of VNREs on positive emotions were mixed across the included studies. 15 studies reported an improvement in positive emotions following exposure to VNREs [45, 46, 48, 49, 51–56, 60, 62–65]. Four of these studies noted that VNREs stimulated the brain's emotional regions through visual and auditory aesthetic experiences, promoting appreciation and evoking a sense of awe [48, 54, 63, 65]. Additionally, Diessner et al. [48] reported that VNREs also induced a feeling of elevation. Smalley et al. [63] found that grand natural landscapes evoked a sense of awe and were associated with feelings of nostalgia. They also observed that adding background music further heightened participants' feelings of excitement.

Four studies indicated that VNREs fostered a sense of connection to the natural environment, thereby enhancing well-being [46, 53–55]. However, Lau et al. [55] noted that while a three-week VNRE intervention improved well-being, the benefits did not persist over time.

Three other studies found that VNREs could guide individuals to experience positive emotions and increase valence [49, 52, 53], suggesting that VNREs enhanced valence by boosting subjective hedonic experiences. However, Jin et al. [52] discovered that the effect on valence was more pronounced in blue spaces compared to green spaces, and the positive impact of VNREs was reduced in individuals with higher levels of depression.

Additionally, three studies reported that watching VNREs helped individuals relax and feel rested, leading to a sense of calmness [49, 62, 63]. One study further indicated that VNREs could effectively allow individuals to restore personal resources, including mood, fatigue, and arousal, during breaks [64].

However, three studies did not find any significant effect of VNREs on positive emotions [28, 53, 59]. Kimura et al. [53] found no changes in arousal levels after watching VNREs. Moreover, another study observed that while VNREs brought participants to a state of calmness, it also reduced their feelings of excitement. Notably, the reduction in positive emotions was less pronounced in blue spaces compared to green spaces [61].

##### Restorativeness

A total of 10 studies reported findings related to the restorativeness of VNREs. These studies used four different scales to assess restorativeness (see Table 7). Nine studies indicated that VNREs could effectively create restorative environments, offering benefits such as a sense of being away, compatibility, extent, and fascination. These factors helped individuals detach from daily stress and avoid excessive mental resources [45, 46, 57, 59–61, 63–65]. Several studies also suggested that the type of natural landscape shown in VNREs could influence the level of restorativeness. For example, green spaces were found to have a higher restorative potential than blue spaces [57], and wild landscapes were more restorative than well-maintained ones [65]. Background audio in VNREs also impacted the restorative experience. VNREs with natural sounds were more restorative than silent ones [61], and natural sounds alone were more effective in enhancing restorativeness than other sound combinations (such as music, music combined with natural sounds, or silence) [63].

However, one long-term study found no significant restorativeness immediately following a three-week VNRE intervention. Improvements in restorativeness were observed only in a follow-up assessment [55].

##### Stress and relaxation

Six studies examined the effect of VNREs on psychological stress [33, 51, 59, 61, 62, 66]. Three studies used stress subscales within mood questionnaires and three studies used context-specific stress scales to evaluate levels of stress (see Table 7). Three of these studies assessed stress levels following stress-inducing tasks [33, 62, 66].

The findings from all six studies indicated that viewing VNREs allowed participants to experience audiovisual stimulation, promoting mental and physical relaxation, capturing their attention, thereby effectively reducing mental stress. This included reductions in emotional stress [51, 59, 62], work-related stress [61], stress-related self-consciousness towards body sensations [33], and exercise-related stress [66]. Further research revealed that background audio in VNREs had a significant impact on stress relief. One study indicated that VNREs with natural sounds were more relaxing than silent VNREs [61]. Another study reported that VNREs accompanied by natural sounds were more effective in reducing stress than those with mechanical sounds or traffic noise [51]. Notably, Meuwese et al. [59] found that the effectiveness of VNREs in reducing stress could be influenced by individual depression levels, with higher levels of depression linked to greater stress-relief effects.

##### Behaviour

Four studies reported on the effects of VNREs on behaviour. Three of these studies examined the influence of VNREs on PEB [48, 50, 54], each using different methods to assess various behaviours related to PEB (see Table 7). Diessner et al. [48] found that viewing VNREs evoked feelings of awe and elevation, which indirectly promoted environmental intentions and led participants to engage in donation behaviours. Ibanez et al. [50] indicated that after the intervention, participants’ sense of connectedness to nature increased, which led to more donations and recycling behaviours, with individuals holding weaker environmental beliefs benefiting the most. Lange et al.[54] conducted two experiments to assess PEB. The first intervention showed an improvement in participants' psychological state, resulting in increased engagement in volunteer activities and PEB. However, the second experiment showed a weaker or nearly absent effect on PEB.

Additionally, one study evaluated the effect of VNREs on procrastination and indicated that after viewing VNREs, participants experienced effective restoration of focus and mental energy, which led to a significant reduction in short-term procrastination among college students [45].

##### Constructive relationships

Four studies investigated the effects of VNREs on constructive relationships [31, 55, 60, 65]. Three of these studies used three different scales to evaluate the effect of VNREs on individuals' sense of natural connectedness (see Table 7) [31, 55, 65]. In a three-week intervention, Lau et al. [55] found that repeated exposure to VNREs gradually strengthened individuals' bond with nature, significantly enhancing their sense of natural connectedness. Yeo et al. [31] reported that viewing VNREs increased participants' sense of presence, which in turn reinforced their sense of natural connectedness. van Houwelingen-Snippe et al. [65] found that VNREs elicited awe and an open mindset, helping participants feel a deeper connection to nature.

Two other studies used various scales to assess the effect of VNREs on the sense of social connectedness, including loneliness, belonging, and social aspirations (see Table 7). Results showed that VNREs, by displaying vast natural landscapes, provided visual stimulation that increased individuals' sense of closeness to nature, thereby reducing feelings of loneliness [60, 65]. Neale et al. [60] also found that viewing VNREs improved participants’ sense of belonging. van Houwelingen-Snippe et al. [65] further assessed the impact on social aspirations and found that exposure to VNREs reduced loneliness and increased social aspirations. The study also found that well-maintained natural landscapes were more effective than wild natural settings in enhancing individuals’ social aspirations.

##### Energy and coping ability

Two studies investigated the effect of VNREs on subjective energy, though the results were mixed [45, 61]. Ojala et al. [61] found that after viewing VNREs featuring blue and green spaces, participants entered a calmer state, with energy levels decreasing, and the decline was more pronounced in green space settings. In contrast, Bielinis et al. [45] observed that VNRE interventions restored participants' focus and attention, reduced fatigue, and subsequently improved subjective energy. Both studies used the Subjective Vitality Scale (SVS) to assess these outcomes.

One study examined coping ability. Meuwese et al. [58] conducted a task inducing serious regret (autobiographical recall) and evaluated coping ability across three trials using the State Coping Scale (SCS). Findings indicated that VNREs helped participants rest and restore directed attention, enabling them to better focus on reflection and problem-solving under psychological stress, thereby enhancing cognitive coping ability. While coping ability significantly improved after the first and the third interventions, the second trial showed an increase only in the coping quantity, with no notable changes in coping quality.

#### Moderating variables

This section examines the moderating variables that influence the effectiveness of VNREs in the included studies. These variables mainly relate to the design of VNREs (e.g. familiarity, multi-sensory experience, degree of naturalness, visual aesthetics) and individual differences among participants (such as depression levels, sensory processing sensitivity, and environmental beliefs).

Ten studies identified the type of natural landscape depicted in VNREs as a critical factor affecting their effectiveness. Among these, four studies compared blue spaces (e.g. oceans, streams, waterfalls) and green spaces (e.g. forests, grasslands, farmland) [52, 57, 61, 62]. However, the findings were not consistent. One study found that green spaces were significantly more effective than blue spaces in promoting restorativeness [57]. Conversely, another study reported that blue spaces were more beneficial for enhancing emotion and valence [52]. Ojala et al. [61] also noted that blue spaces had a smaller impact on reducing positive emotions and subjective energy. Meanwhile, Reece et al. [62] observed no significant differences in the positive effects of blue and green spaces on positive or negative emotions. Additionally, one study explored the impact of different types of green spaces and found that VNREs depicting forest landscapes had a significantly positive effect on well-being, while farmland landscapes did not produce similar results [46].

Five studies examined how the characteristics of natural landscapes affect the effectiveness of VNREs [48, 49, 51, 60, 65]. One study investigated the impact of familiarity with the natural landscape, finding that both familiar local landscapes and unfamiliar distant landscapes improved emotion and reduced stress [51]. Two studies investigated the degree of naturalness in VNRE landscapes and its effect on intervention outcomes [60, 65]. One study found no significant difference in the sense of social connectedness, restorativeness, or emotional impact between landscapes with or without people [60]. In contrast, van Houwelingen-Snippe et al. [65] reported that managed landscapes elicited greater social expectations, while wild landscapes provided stronger restorativeness.

Finally, two studies examined the relationship between the visual aesthetics of VNREs and their intervention outcomes [48, 49]. One study found that awe-inspiring natural landscapes (such as forests and oceans) were more effective in evoking awe and promoting PEB than ordinary landscapes (such as deserts) [48]. Another study found a positive correlation between the pleasantness of the VNRE scene and participants’ feelings of pleasure, with pleasing landscapes (such as jungle sunsets) providing a stronger sense of enjoyment than neutral landscapes (such as flowing rivers) and unpleasant landscapes (such as glacier calving) [49].

Four studies examined the effects of VNREs under different sensory conditions [51, 61, 63, 64]. Three studies focused on auditory conditions and found that VNREs with rich auditory elements were significantly more effective than silent environments in improving emotion, enhancing restorativeness, and reducing stress [51, 61, 63]. However, Jiang et al. [51] noted that VNREs with background mechanical and traffic noise had a more negative impact on emotion and stress. Additionally, two studies further analysed the effects of different types of auditory stimuli. Smalley et al. [63] found that, compared to natural sounds alone, a combination of natural sounds and music did not significantly enhance restorativeness, calmness, a sense of awe, or nostalgia but did increase feelings of excitement. Music alone also showed a significant effect only in boosting feelings of excitement. Similarly, Jiang et al. [51] reported that natural sounds were more effective in relieving stress than mechanical and traffic noise. Sona et al. [64] conducted a study on the influence of olfactory stimuli, finding that adding scents matched to the natural scene significantly enhanced resource restoration and the positive restorative experience, compared to VNREs without scent.

Four studies investigated the influence of individual differences on the mental health effects of VNREs, covering factors such as depression levels, sensory processing sensitivity, and environmental beliefs [47, 50, 52, 59]. Two studies yielded mixed findings on the impact of depression levels on intervention effectiveness [52, 59]. One study found that individuals with higher levels of depression experienced less improvement in emotion from VNREs [52]. In contrast, another study suggested that higher depression levels enhanced the effects of VNREs in reducing negative emotions, promoting restorativeness, and alleviating stress [59]. Additionally, Cadogan et al. [47] examined the role of sensory processing sensitivity, finding that individuals with higher sensitivity experienced more significant improvements in negative emotions after viewing VNREs. Lastly, Ibanez et al. [50] investigated the effect of environmental beliefs on PEB, showing that individuals with lower environmental beliefs exhibited a significant increase in PEB after watching VNREs.

## Discussion

### Application of VNREs

This review examined the application of VNREs among the general adult population. The results indicated that VNREs effectively promoted mental health in both in-person intervention and remote online interventions. This finding aligns with previous research that demonstrated the efficacy of video interventions for mental health both online and offline [69, 70]. However, the results also revealed differences in intervention effectiveness between online environments and laboratory settings under similar conditions [59]. Some studies suggested that participants in online interventions might be more susceptible to external distractions compared to those in offline environments, which could impact intervention outcomes [71]. Future research should further investigate the effects of different intervention settings, such as exploring strategies to minimize external distractions in online environments.

This review also demonstrated that VNREs had positive mental health effects across various devices, regardless of whether the intervention duration was long or short. These devices included both public screens and personal mobile devices. Previous research supported this finding, indicating that even brief simulated nature experiences could evoke positive emotions and restorativeness [72] and that nature-based experiences could also be achieved through personal mobile devices [35]. However, some studies pointed to limitations of mobile devices in terms of psychological restoration [73], and both screen size and intervention duration were considered key factors affecting the mental health benefits of simulated nature [74–76]. Future research should explore the potential impact of device type, screen size, and intervention duration on the effectiveness of VNREs.

This review also integrated findings on the application of video content and background audio in VNREs. Results indicated that most VNREs promoted mental health by presenting green spaces with natural sounds (e.g., forests, grasslands) or blue spaces (e.g., oceans, rivers). Consistent with previous research, simulated blue and green environments, along with their natural sounds, had a significant positive impact on mental health [77–79]. This review indicated that, in addition to standalone natural sounds, combinations of natural sounds had positive mental health effects. For example, adding music to VNREs effectively enhanced feelings of excitement [63]. Previous research suggested that powerful background music could increase the impact of nature videos and capture viewers' attention [80]. This implies that background audio design in VNREs could be a promising area for further research. Future studies should explore how to optimize background audio design to enhance the mental health benefits of VNREs.

### Effects of VNREs on mental health

This review synthesized findings on the general adult population's mental health effects of VNREs. Most studies indicated that VNREs had positive effects on emotions, restorativeness, stress and relaxation, behaviour (PEB and procrastination), constructive relationships (the sense of natural and social connectedness), energy, and coping ability. Previous research supported these findings, showing that simulated restorative environments could effectively enhance emotions [69, 81], restorativeness [82], stress and relaxation [83], behaviour (PEB and procrastination) [84, 85], constructive relationships (sense of natural and social connectedness) [86, 87], and energy [82]. Notably, compared to existing literature, this review presents new and relatively rare insights into the effects of VNREs on behaviour, constructive relationships, and coping ability. To our knowledge, this review is the first to reveal the potential of simulated nature environments in enhancing cognitive coping ability. Future studies should further explore the role of VNREs in these areas to strengthen the evidence base.

Further analysis revealed that the mental health benefits of VNREs were mostly short-term and might gradually diminish after the intervention ended. Some research suggested that short-term psychological interventions inherently had limitations, with effects that would decrease over time [88]. This indicated certain limitations in VNREs' ability to sustain long-term mental health benefits. Nonetheless, brief breaks themselves were valuable resources for restoration [89, 90] and served as an important public health tool [22]. Future efforts should focus on optimizing VNRE design to extend their mental health benefits. For example, embedding VNREs frequently in daily environments could help prevent the accumulation of psychological stress, thereby providing long-term positive effects.

This review also found disagreements regarding the effectiveness of VNREs on emotion, energy, and behaviour. Similar observations were reported in previous reviews [38, 91]. Regarding emotion and energy, some research indicated that virtual nature could alleviate negative emotions, such as calmness and relaxation, typically through brief rest periods [92]. According to the Three Circle Model of Emotion, this calming experience could reduce excitement, pleasure, and energy [93], whereas the activation of positive emotions and energy might require deeper interaction between the individual and the environment [94, 95]. As a result, some studies reported suboptimal or even negative effects on the improvement of emotion and energy. Behavioural inconsistencies were primarily observed in PEB outcomes. Further analysis suggested that these discrepancies might be due to differences in types of PEB, such as donations [48], recycling [50], and environmental protection [54]. Different types of behaviours might engage distinct psychological mechanisms and assessment methods, leading to varied results [96]. Overall, the differences in underlying mechanisms and evaluation methods for VNREs’ mental effects led to inconsistent findings. Future research should focus on examining the mechanisms behind VNREs' mental health impacts and refining categories of influence to better assess their mental health benefits. For example, using qualitative or mixed-methods approaches could provide a more comprehensive understanding of VNREs' effects on mental health.

Additionally, some included studies reported inconsistent results across multiple experiments under the same intervention conditions, particularly in areas such as PEB [54], coping ability [58], and negative emotions [59]. Analysis of the quality assessment and risk of bias revealed certain limitations in these studies' experimental designs, such as insufficient sample sizes (e.g., lack of power analysis, overly small sample sizes) [58], sample heterogeneity (e.g., significant background differences among sample groups) [54], and variation in experimental conditions (e.g., different video conditions used as control groups and varying tasks for emotion induction) [54, 59]. Previous research suggested that lack of power analysis, inconsistent control group designs, and differing induction methods could affect the accuracy and comprehensiveness of results [97, 98]. This indicated that the mental health effects of VNREs could be influenced by experimental design to a certain extent. Therefore, future studies should adopt more rigorous and scientifically sound experimental methodologies to improve the validity and reliability of their findings, such as conducting comprehensive data analysis and refining the control group design.

### Moderating variables

This review conducted a comprehensive analysis of the moderating variables reported in the included studies. The moderating variables involved aspects of VNRE intervention design (video content, familiarity, degree of naturalness, visual aesthetics, and multi-sensory components: auditory and olfactory), and individual differences among participants (depression levels, sensory processing sensitivity, and environmental beliefs). This aligns with previous research suggesting that variables like natural landscape [99], multi-sensory engagement (olfactory [100], auditory [30]), degree of naturalness [101], depression level [102], sensory processing sensitivity [103], attitudes toward nature [104], and visual aesthetics [105] could influence the mental health impact of both simulated and real natural environments. Although results showed that familiarity with video content did not affect VNREs' effect on emotion and stress [51], other research indicated that familiarity could play a role in perceived restorativeness [106]. Therefore, further research is needed to clarify the role of familiarity in the effectiveness of VNREs. Additionally, findings on the relative effectiveness of blue and green environments for mental health remain inconsistent. Beyond environmental characteristics, individual differences could also affect intervention outcomes; for instance, natural water scenes could evoke anxiety or unease in some individuals [107]. Future research should further investigate the impact of individual differences on the effectiveness of blue and green space in VNREs to enhance the precision of these interventions.

Additionally, the results showed inconsistencies in how individual depression levels influence the effectiveness of VNRE interventions. One study found a negative correlation, suggesting that higher depression levels weakened the intervention’s effect [52], but another study observed the opposite effect [59]. Further analysis revealed differences in the degree of naturalness in the VNREs used: [52] used a video of a more complex, human-modified park landscape, while [59] used a video of more nature. The human-modified scene might impose additional cognitive load, which could hinder the restorative process, especially for individuals with depression, who are known to struggle more with complex tasks [108]. This suggested that the level of naturalness in VNREs might affect the mental health recovery of individuals with depression. Furthermore, results also showed mixed effects of naturalness on the intervention outcomes. Some studies indicated that a higher degree of naturalness in VNREs enhanced restorativeness and constructive relationships [61, 63, 65], consistent with prior findings [101, 109, 110]. However, the results suggested that the presence of people does not necessarily diminish the restorative or social relationship benefits of VNREs [60]. Some research even proposed that human presence can foster social interaction, enhancing the health benefits of green environments [111]. This implies that, in certain contexts, the involvement of people did not reduce the naturalness or psychological benefits of VNREs. Despite these insights, there remains a lack of in-depth research on factors influencing the naturalness of VNREs and their role in mental health recovery. Future research should investigate how specific factors affect VNRE naturalness and examine their distinct contributions to individual mental health recovery.

Moreover, previous research identified additional variables that could influence the mental health effects of simulated or real nature, such as species richness (number and diversity of plant species) [112], intervention duration [74, 76], screen size [75], socio-demographic factors [113], personal preferences [114], sense of place [115], social context [106], and perceived safety [106]. These findings indicated that the effectiveness of VNREs on mental health could be influenced by a broad range of factors. Future research should continue to explore these variables to enhance the potential of VNRE interventions for mental health improvement.

### VNREs and real natural environments

The results revealed debate over whether VNREs could fully replace real outdoor nature exposure [28, 62]. Similar issues arose in previous research. While simulated natural environments could offer certain benefits [22, 116], they consistently fell short of replicating the effects of real nature [117, 118]. Some researchers attributed this to the inability of simulated environments to provide the multi-sensory experiences of real nature, such as fresh air [119] and authentic sensory interaction [120]. Thus, while VNREs could alleviate some challenges associated with limited access to nature, they might not be able to fully substitute for real nature or mitigate the public health drawbacks of urbanization and reduced green environments. Therefore, broader interventions might be needed to fundamentally enhance public mental health and improve public health infrastructure, such as increasing green space in communities. Additionally, further exploration is needed to reduce the gap between VNREs and real nature by enhancing the multi-sensory aspects of VNREs to optimize their role in mental health promotion.

Although VNREs might not be able to entirely replace real nature, their benefits for mental health could still be valuable. Studies suggested that even low-immersion visualizations of nature promoted some level of restoration [121]. While not a complete substitute for real nature, VNREs could complement natural exposure [122], especially for individuals who lack access to real natural environments. Therefore, VNREs remain an effective tool for mental health intervention, helping to enhance public mental health and overall public health outcomes.

## Limitations

This review had some limitations. First, it focused on the general adult population and did not include studies specifically targeting sensitive groups. However, other research showed that simulated nature could also benefit groups such as the elderly [123], children [124], and patients [125]. Future studies should further explore the potential mental health or clinical benefits of VNREs for these special populations.

Additionally, this review emphasized the effect of VNREs on mental health, focusing on psychological aspects defined by the APA Dictionary of Psychology [37], such as emotions, behaviour, anxiety, disabling symptoms, constructive relationships, coping abilities, and stress, with particular attention to restorativeness. Also, this review only focused on outcomes not measured by biological markers. However, other research indicated that simulated restorative environments could also positively impact cognition [104, 124], physiology [126], creativity [127], and mental health issues [128], which are closely related to mental health. Future research could examine VNREs' potential benefits across these areas, providing more comprehensive evidence for public health improvement.

Moreover, several primary studies included in this review had inherent limitations, such as sample-related issues (e.g., selection bias, large sample heterogeneity) and methodological risks (e.g., concerns regarding the randomization process and data loss), which might reduce the reliability of experimental outcomes and introduce bias into the review’s conclusions. Therefore, future studies should adopt more rigorous experimental designs to minimize these factors' impact on results.

Furthermore, the screening of studies was conducted by a single author (DRH), rather than being performed independently by both authors. This may have introduced the potential for bias in the study selection process. Future systematic reviews could mitigate this limitation by involving multiple independent reviewers during the screening phase, which would help reduce the risk of bias and enhance the reliability of study inclusion decisions.

Finally, this systematic review was not pre-registered on platforms such as PROSPERO or similar registries. Future reviews should adhere to standardized protocols, including pre-registration, to ensure compliance with systematic review guidelines.

## Conclusion

This study systematically reviewed studies from the past six years on the impact of VNREs on the mental health of the general adult population, integrating findings on VNRE application and their effects on mental health. To our knowledge, this is the first systematic review specially focused on the mental health effects of VNESs. The results indicated that VNREs provided effective mental health interventions for the general adult population, applicable both online and offline across various devices in both short- and long-term contexts. VNREs showed significant potential for applications in emotional regulation, stress relief, and enhancing restorativeness. Additionally, this study offered new insights into VNREs' effects on behaviour (procrastination and PEB), constructive relationships (sense of natural and social connectedness), and coping abilities (cognitive coping), which could merit further investigation. This review also summarized moderating factors that could influence the mental health effects of VNREs, identifying intervention design elements (video content, familiarity, degree of naturalness, visual aesthetics, multi-sensory components: auditory olfactory) and individual differences (depression levels, sensory processing sensitivity, and environmental beliefs) as influential variables. Although VNREs might not be able to replace real nature, they could serve as valuable mental health interventions for individuals with limited access to natural environments. Future research should further explore the relationship between intervention factors and mental health outcomes, improving intervention design to enhance and sustain the positive effects of VNREs on mental health. This review provided new perspectives and strategic support for mental health interventions in public health, not only detailing VNRE applications among the general adult public but also validating their potential in mental health promotion. This review offered specific recommendations for VNRE development and application, deepening the understanding of simulated nature experiences within the field of public health, offering theoretical support and practical guidance for individuals and organizations interested in exploring these interventions.

## Data Availability

The datasets generated during and/or analysed during the current study are available from the corresponding author upon reasonable request.

## Abbreviations

VNRE: Video-based natural restorative environment
2D: Two-dimensional
VR: Virtual Reality
RCT: Randomized controlled trial
QE: Quasi-experiment
SEQ: Subjective evaluation questionnaire
BTM: Behavioural task measure
RoB 2: Risk of Bias 2
ROBINS-I: Risk of Bias in Non-randomized Studies of Interventions
PEB: Pro-environmental behaviour

## Declarations

Ethics approval and consent to participate.

Not applicable.
